# Muscle contributions to pre-swing biomechanical tasks influence swing leg mechanics in individuals post-stroke during walking

**DOI:** 10.1186/s12984-022-01029-z

**Published:** 2022-06-03

**Authors:** Lydia G. Brough, Steven A. Kautz, Richard R. Neptune

**Affiliations:** 1 Walker Department of Mechanical Engineering, The University of Texas at Austin, Austin, TX USA; 2 Ralph H Johnson VA Medical Center, Charleston, SC USA; 3 Department of Health Sciences & Research and Division of Physical Therapy, Medical University of South Carolina, Charleston, SC USA; 4 Walker Department of Mechanical Engineering, The University of Texas at Austin, 204 E. Dean Keeton Street, Stop C2200, Austin, TX 78712-1591 USA

**Keywords:** Stiff knee gait, Modeling, Gait, Compensation, Biomechanics

## Abstract

**Background:**

Successful walking requires the execution of the pre-swing biomechanical tasks of body propulsion and leg swing initiation, which are often impaired post-stroke. While excess rectus femoris activity during swing is often associated with low knee flexion, previous work has suggested that deficits in propulsion and leg swing initiation may also contribute. The purpose of this study was to determine underlying causes of propulsion, leg swing initiation and knee flexion deficits in pre-swing and their link to stiff knee gait in individuals post-stroke.

**Methods:**

Musculoskeletal models and forward dynamic simulations were developed for individuals post-stroke (n = 15) and healthy participants (n = 5). Linear regressions were used to evaluate the relationships between peak knee flexion, braking and propulsion symmetry, and individual muscle contributions to braking, propulsion, knee flexion in pre-swing, and leg swing initiation.

**Results:**

Four out of fifteen of individuals post-stroke had higher plantarflexor contributions to propulsion and seven out of fifteen had higher vasti contributions to braking on their paretic leg relative to their nonparetic leg. Higher gastrocnemius contributions to propulsion predicted paretic propulsion symmetry (p = 0.005) while soleus contributions did not. Higher vasti contributions to braking in pre-swing predicted lower knee flexion (p = 0.022). The rectus femoris had minimal contributions to lower knee flexion acceleration in pre-swing compared to contributions from the vasti. However, for some individuals with low knee flexion, during pre-swing the rectus femoris absorbed more power and the iliopsoas contributed less power to the paretic leg. Total musculotendon work done on the paretic leg in pre-swing did not predict knee flexion during swing.

**Conclusions:**

These results emphasize the multiple causes of propulsion asymmetry in individuals post-stroke, including low plantarflexor contributions to propulsion, increased vasti contributions to braking and reliance on compensatory mechanisms. The results also show that the rectus femoris is not a major contributor to knee flexion in pre-swing, but absorbs more power from the paretic leg in pre-swing in some individuals with stiff knee gait. These results highlight the need to identify individual causes of propulsion and knee flexion deficits to design more effective rehabilitation strategies.

**Supplementary Information:**

The online version contains supplementary material available at 10.1186/s12984-022-01029-z.

## Background

Over 795,000 people in the United States experience a stroke each year and over half of individuals post-stroke over age 65 have reduced mobility [1]. Regaining walking ability is an important goal of rehabilitation as walking speed is a critical predictor of long-term health [2] and individuals post-stroke who achieve limited or full community walking speeds report an overall higher quality of life than those who remain household ambulators [3]. Successful walking requires the execution of the critical pre-swing biomechanical subtasks of body propulsion and leg swing initiation, which are often impaired post-stroke [4, 5] and may influence swing phase knee flexion [2, 6–8].

For example, modeling studies having identified knee flexion velocity at toe-off as the primary contributor to peak knee flexion during swing [9] and low push-off acceleration has also been linked to stiff knee gait [2]. Moreover, impaired knee flexion is often attributed to rectus femoris activity [10, 11] and a modeling study showed that eliminating rectus femoris activity in pre-swing was more effective than eliminating rectus femoris activity in early swing for improving knee flexion [7]. Decreased gastrocnemius activity may also contribute to stiff knee gait, as increased gastrocnemius contributions to pre-swing knee flexion were observed after gait retraining [8]. However, a representative individual post-stroke with a limited community walking speed had lower iliopsoas contributions to leg swing initiation in pre-swing but similar contributions from the gastrocnemius compared to a healthy control [12]. Thus, while the potential of lower extremity muscles to increase or decrease knee flexion velocity in late stance has been documented [13], it is unknown which muscles most affect pre-swing knee flexion velocity in individuals post-stroke.

Braking and propulsion deficits are also common in individuals post-stroke [4], and in addition to predicting slower walking speeds [14, 15], may contribute to stiff knee gait. The plantarflexors are primary contributors to propulsion [16, 17]. Decreased plantarflexor contributions to propulsion have been observed in individuals post-stroke [12, 18, 19], which could occur due to both muscle activation deficits [20, 21] and altered muscle and Achilles tendon properties [22–24]. The gastrocnemius is an important contributor to both propulsion and leg swing initiation [17], and thus low propulsion may be related to knee flexion deficits. Stimulating the plantarflexors in pre-swing increases peak knee flexion for individuals post-stroke [25]. However, the total propulsive force did not predict knee flexion [6]. On average, individuals with impaired plantarflexor coordination do not have lower propulsion, but rather greater braking [26] likely due to co-activation of the plantarflexors and vasti muscles, which are primary contributors to braking [9]. Knee flexion velocity at toe-off may be diminished by late braking forces because muscles such as the vasti and rectus femoris that contribute to braking also contribute to knee extension and oppose leg swing initiation in late-stance [9]. In addition, late stance braking forces in individuals post-stroke predict less knee flexion during swing [6]. However, it is unknown if the relationships between braking, propulsion and swing phase kinematics are causal or correlative. Due to the characteristically high variability between individuals post-stroke, there are a number of mechanisms that can cause both propulsion and knee flexion deficits.

Previous work has established the importance of pre-swing conditions to achieving adequate swing phase knee flexion. However, actual muscle contributions to propulsion, knee velocity and leg-swing initiation in individuals post-stroke and their relationship to swing-phase knee flexion has not been established. Thus, the objectives of this study were to determine the underlying causes of propulsion and braking deficits and identify muscle contributors to pre-swing knee flexion acceleration and leg swing initiation in individuals post-stroke with and without stiff knee gait. We hypothesized that (1) braking and propulsion asymmetries would be caused by both low plantarflexor contributions to propulsion and high vasti contributions to braking, (2) vasti and plantarflexor contributions to propulsion and braking in pre-swing would predict swing phase knee flexion, (3) the rectus femoris would be a major contributor to knee extension in pre-swing in individuals with stiff knee gait, and (4) total musculotendon power delivered to the leg in pre-swing would predict knee flexion during swing. The outcomes of this work will highlight specific deficits in propulsion and leg swing initiation post-stroke and their implications for swing phase knee flexion, which will provide a basis for developing targeted rehabilitation strategies.

## Methods

### Data collection

Kinematic, kinetic and electromyography data were collected from 15 individuals post-stroke (6 female, age: 56.1 ± 13.3 years, at least six months post-stroke) and 5 age-similar control subjects (2 female, age: 53.4 ± 7.3 years) (Table 1). Participants provided informed written consent to this Institutional Review Board approved protocol. Participants walked on a split-belt instrumented treadmill (Bertec, Columbus, OH) at their self-selected walking speed without the use of assistive devices. Before data collection was initiated, participants practiced treadmill walking to get comfortable with the experimental setup and walked for at least 10 s to reach steady-state before each 30-s trial. Kinematic data were collected at 120 Hz using a 12-camera motion capture system and 65 active markers (PhaseSpace, San Leandro, CA). Electromyography (EMG) data were collected (Motion Labs, Cortlandt, NY) at 1000 Hz from bilateral electrodes placed on the medial gastrocnemius, soleus, vastus medialis, lateral hamstrings, medial hamstrings, rectus femoris and tibialis anterior. Kinematic and ground reaction force (GRF) data were low-pass filtered at 6 Hz and 15 Hz, respectively. EMG data were high-pass filtered at 40 Hz, demeaned, rectified and low-pass filtered at 4 Hz.

**Table 1:** Participant characteristics and clinical scores for the Fugl Meyer Lower Extremity (FM LE), Dynamic Gait Index (DGI) Six Minute Walk Test (6MWT), and over ground (OG) self-selected walking speed

Individuals post-stroke										
Participant	Age (yrs)	Mass (kg)	Height (m)	Treadmill self-selected walking speed (m/s)	Sex	Months since stroke	FM LE	DGI	6MWT (m)	OG walking speed (m/s)
1	75	66.6	1.61	0.44	M	–	31	21	418	1.08
2	67	76.1	1.54	0.55	F	–	28	16	347	0.99
3	58	76.8	1.59	0.55	F	21	29	22	415	0.90
4	51	85.9	1.69	0.55	M	–	31	21	410	0.98
5	53	112.7	1.78	0.35	F	28	21	18	329	0.79
6	63	114.6	1.68	0.40	M	54	26	–	–	0.91
7	49	93.5	1.92	0.40	M	19	24	19	312	1.18
8	70	85.0	1.82	0.30	M	47	25	15	236	0.68
9	70	86.4	1.80	0.30	M	29	24	19	257	0.88
10	55	53.7	1.60	0.30	F	81	16	13	218	0.36
11	60	75.8	1.68	0.45	M	26	23	18	260	0.75
12	35	63.7	1.58	0.50	F	21	20	18	346	0.70
13	66	98.5	1.80	0.40	M	127	19	–	–	0.65
14	26	77.7	1.62	0.30	F	33	23	15	229	0.38
15	43	84.9	1.69	0.20	M	–	9	12	262	0.64
Average	56 ± 13	83.5 ± 16.2	1.69 ± 0.11	0.4 ± 0.1		44 ± 33	23 ± 6	17 ± 3	311 ± 73	0.79 ± 0.23

Control participants					
Participant	Age (yrs)	Mass (kg)	Height (m)	OG walking Speed (m/s)	Sex
C1	59	81.3	1.74	0.55	M
C2	40	79.7	1.68	1.10	M
C3	51	83.6	1.58	0.70	F
C4	59	80.1	1.71	0.50	M
C5	58	65.3	1.50	0.80	F
Average:	53 ± 7	78.0 ± 6.5	1.64 ± 0.10	0.7 ± 0.2	

Note that months since stroke and clinical scores for DGI and 6MWT were not available for all participants

### Musculoskeletal models and simulations

A representative paretic leg gait cycle (left gait cycle for control subjects) for each participant was chosen for further analysis using the functional medial distance depth method [27]. Using OpenSim 3.3 [28], a musculoskeletal model [29] with 23 degrees of freedom and 92 Hill-type musculotendon actuators consisting of active and passive elastic elements [30] was scaled to match the anthropometry of each participant. An inverse kinematics analysis estimated generalized coordinates during the selected gait cycle by minimizing the difference between experimental and model markers [28]. To reduce dynamic inconsistencies between the experimental GRFs and body segment kinematics, a residual reduction algorithm (RRA) fine-tuned the torso center of mass (COM) position, segment masses and joint kinematics [28] until residual forces and tracking errors were within acceptable ranges [31]. Computed muscle control (CMC) [32] then estimated muscle excitations that reproduced the experimentally measured motion while minimizing the sum of excitations squared. Muscle excitation timing was constrained to approximately follow normalized EMG signals [33].

### Data analysis

Body propulsion and braking were calculated from the integral of the anterior and posterior GRF, respectively. Propulsion and braking asymmetries were defined as the percentage of paretic propulsion (PP) and paretic braking (PB), i.e., the paretic propulsive or braking impulse divided by the sum of paretic and nonparetic propulsive or braking impulses (e.g., perfectly symmetric PP = 0.5). Heel strike and toe-off were identified from the vertical GRF using a threshold of 20 N. Knee flexion velocity at toe-off and peak knee flexion during swing were identified using joint kinematics from RRA. Pre-swing was defined as the double support phase between nonparetic (right) heel strike and paretic (left) toe-off. Individuals were classified as having stiff knee gait if their peak paretic knee flexion during swing was at least 15° less than their peak nonparetic knee flexion [34, 35].

### Muscle contributions to biomechanical subtasks

Induced acceleration and segmental power analyses [36] were used to determine individual muscle contributions to braking, propulsion, knee flexion in pre-swing and leg swing initiation. Muscle contributions were then analyzed in functional groups (Table 2). To perform the induced acceleration analyses, a surface rolling constraint was applied to the feet during stance [37] and muscle forces were determined using activations from CMC. Results were compared to experimental GRFs to ensure that the acceleration of the COM tracked the normalized GRFs. Muscle contributions to braking and propulsion were defined as each muscle’s contribution to the anteroposterior (AP) acceleration of the body’s COM integrated with respect to time over stance, normalized by walking speed. Muscle contributions to knee flexion during pre-swing were determined by integrating each muscle’s contribution to knee flexion acceleration over time. To determine muscle contributions to leg swing initiation, a segment power analysis was used to determine the power delivered, absorbed or transferred to the leg by each muscle [9]. Musculotendon power was integrated over time to determine each musculotendon unit’s total work on the leg during pre-swing and was analyzed with and without normalizing by walking speed. Results were normalized by walking speed because walking speed predicts AP GRFs, knee flexion, and musculotendon power. However, results for musculotendon work were also presented without normalization so the reader can interpret absolute work in addition to work relative to walking speed.

**Table 2:** Muscle analysis groups

Muscle group	Muscles
IL	Iliacus, psoas
AL	Adductor longus, adductor brevis, pectineus, quadratus femoris
AM	Superior, middle, inferior adductor magnus
SAR	Sartorius
RF	Rectus femoris
VAS	Vastus medialis, vastus intermedius, vastus lateralis
GMEDA	Anterior and middle gluteus medius, anterior and middle gluteus minimus
GMEDP	Posterior gluteus medius, posterior gluteus minimus
TFL	Tensor fasciae latae
GMAX	Superior, middle and inferior gluteus maximus
HAM	Semimembranosus, semitendinosus, biceps femoris long head, gracilis
BFSH	Biceps femoris short head
GAS	Medial gastrocnemius, lateral gastrocnemius
SOL	Soleus, tibialis posterior, flexor digitorum longus
TA	Tibialis anterior, extensor digitorum longus

### Statistical analyses

To test the hypotheses that greater vasti contributions to braking and lower plantarflexor contributions to propulsion would predict braking and propulsion asymmetries, linear regression models were created with PP and PB as the dependent measures and either soleus, gastrocnemius or vasti contributions to AP COM acceleration over stance as the independent measure. To test the hypothesis that pre-swing braking and propulsion would predict swing-phase knee flexion, linear regression models were created with peak knee flexion as the dependent measure and either total pre-swing AP GRF impulse (normalized by subject mass and walking speed), soleus, gastrocnemius or vasti contributions to AP COM acceleration in pre-swing (normalized by walking speed) as the independent measures. Similarly, to test the hypothesis that individuals with low knee flexion would have less paretic musculotendon work performed on the paretic leg, a linear regression was created with peak knee flexion as the dependent measure and net musculotendon work performed on the paretic leg as the independent measure. Linear regressions were created with one predictor and one outcome measure at a time using the linear regression model tool in MATLAB (Mathworks, Natick, MA). Significance was defined as α = 0.05.

## Results

### Predictors of braking and propulsion asymmetry

**Fig. 1:**
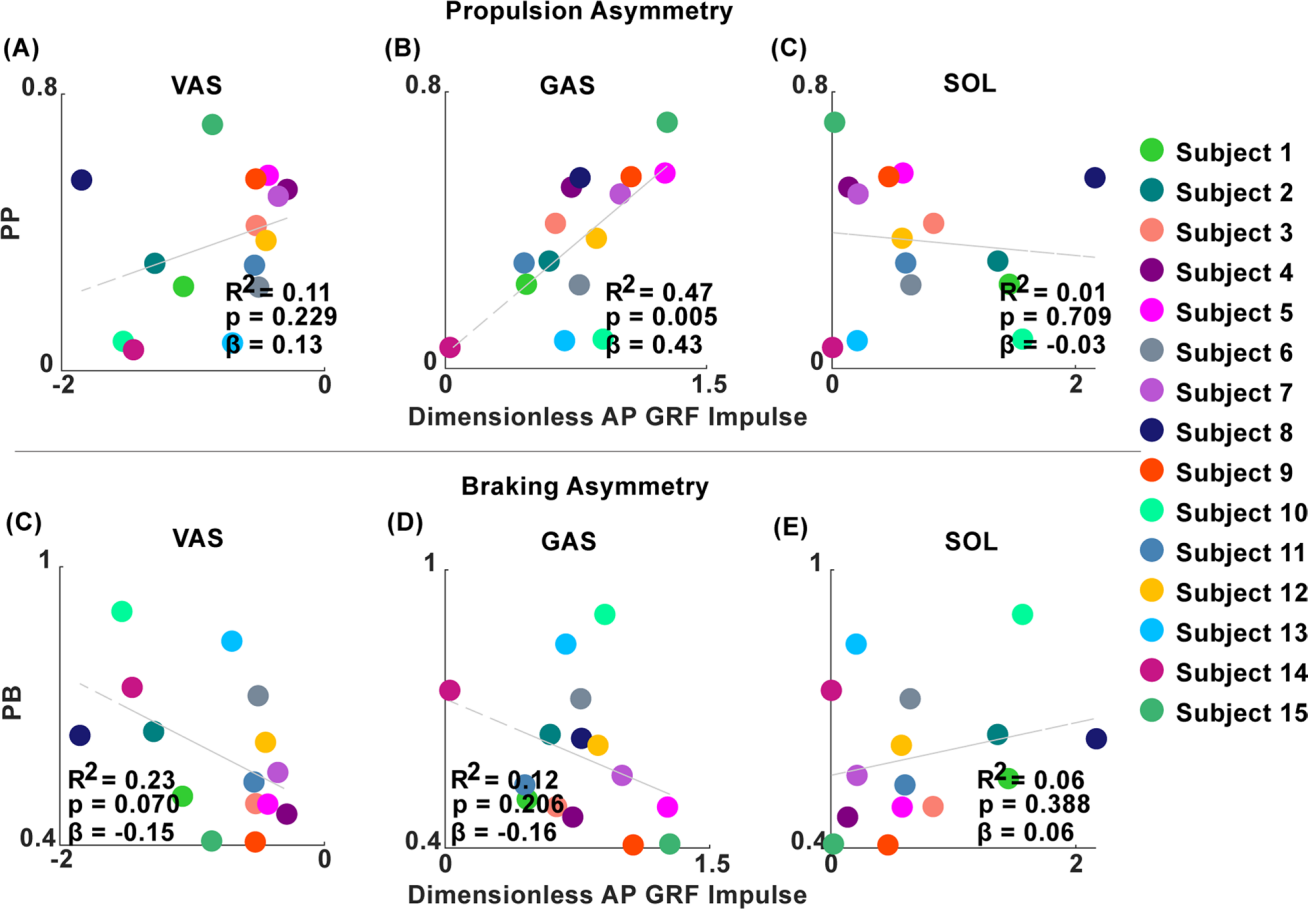
Significant and non-significant predictors of propulsion and braking asymmetry. Percentage paretic propulsion (PB) and percentage paretic braking (PB) vs. muscle contributions to propulsion and braking in individuals post-stroke

**Fig. 2:**
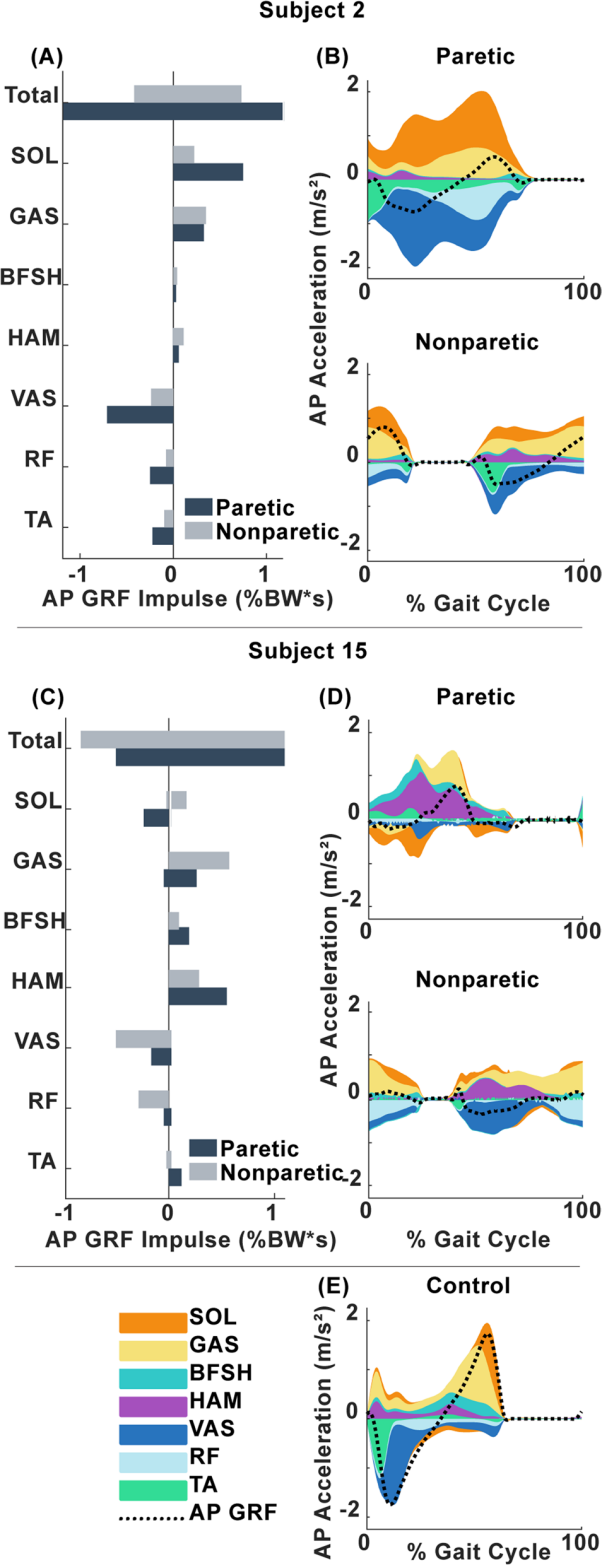
Muscle contributions to braking and propulsion*.* **A** Paretic and nonparetic muscle contributions to AP COM acceleration integrated over stance for Subject 2. **B** Muscle contributions to AP COM acceleration over the paretic gait cycle, with contributions stacked on top of one another and shown relative to the normalized AP GRF (dotted line) for Subject 2. **C** Paretic and nonparetic muscle contributions to AP COM acceleration integrated over stance for Subject 15. **D** Muscle contributions to AP COM acceleration over the paretic gait cycle, with contributions stacked on top of one another and shown relative to the normalized AP GRF (dotted line) for Subject 15. **E** Muscle contributions to AP COM acceleration over the left gait cycle, with contributions stacked on top of one another and shown relative to the normalized AP GRF (dotted line) for a representative control subject

Neither vasti nor soleus contributions to propulsion predicted propulsion asymmetry (Fig. 1A and C) but gastrocnemius contributions did predict propulsion asymmetry (Fig. 1B) (p = 0.005, R^2^ = 0.47, β = 0.43). Vasti, gastrocnemius and soleus contributions to AP GRFs did not predict braking asymmetry (Fig. 1D–F). Four out of fifteen individuals post-stroke had greater plantarflexor contributions to propulsion on their paretic leg compared to their nonparetic leg (see Additional file 1: Table S3). All four produced more braking with the paretic vasti than the nonparetic vasti. A total of seven out of fifteen individuals post-stroke produced more braking with the paretic vasti than the nonparetic vasti. Two examples of muscles contributions to braking and propulsion are provided to illustrate propulsion asymmetries in opposite directions (Fig. 2).

### Braking and propulsion predictors of swing phase knee flexion

**Fig. 3:**
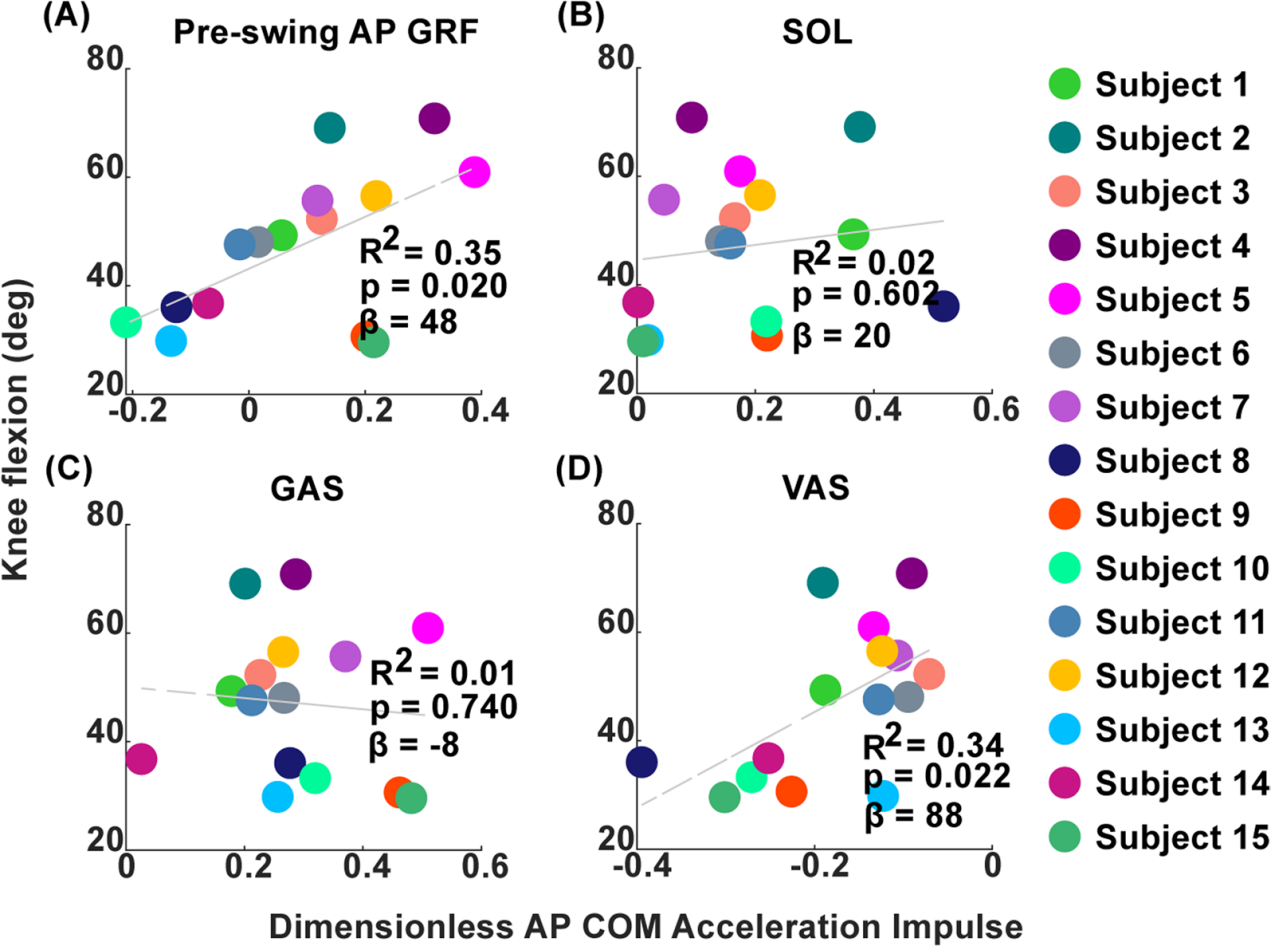
Significant and non-significant predictors of peak knee flexion. Peak knee flexion during swing vs. potential predictors of knee flexion, including **A** the impulse of paretic AP GRFs in pre-swing normalized by subject mass, **B**–**D** The AP COM acceleration impulse in pre-swing contributed by the SOL, GAS and VAS groups

Greater pre-swing AP GRF impulse was a significant predictor of greater peak swing phase knee flexion (Fig. 3A) (p = 0.02, R^2^ = 0.35, β = 48). Lower vasti contributions to braking in pre-swing relative to walking speed also predicted greater peak knee flexion during swing (Fig. 3D) (p = 0.02, R^2^ = 0.35, β = 88) while soleus and gastrocnemius contributions to propulsion in pre-swing did not (Fig. 3B and C).

### Primary contributors to knee flexion and extension acceleration in pre-swing

**Fig. 4:**
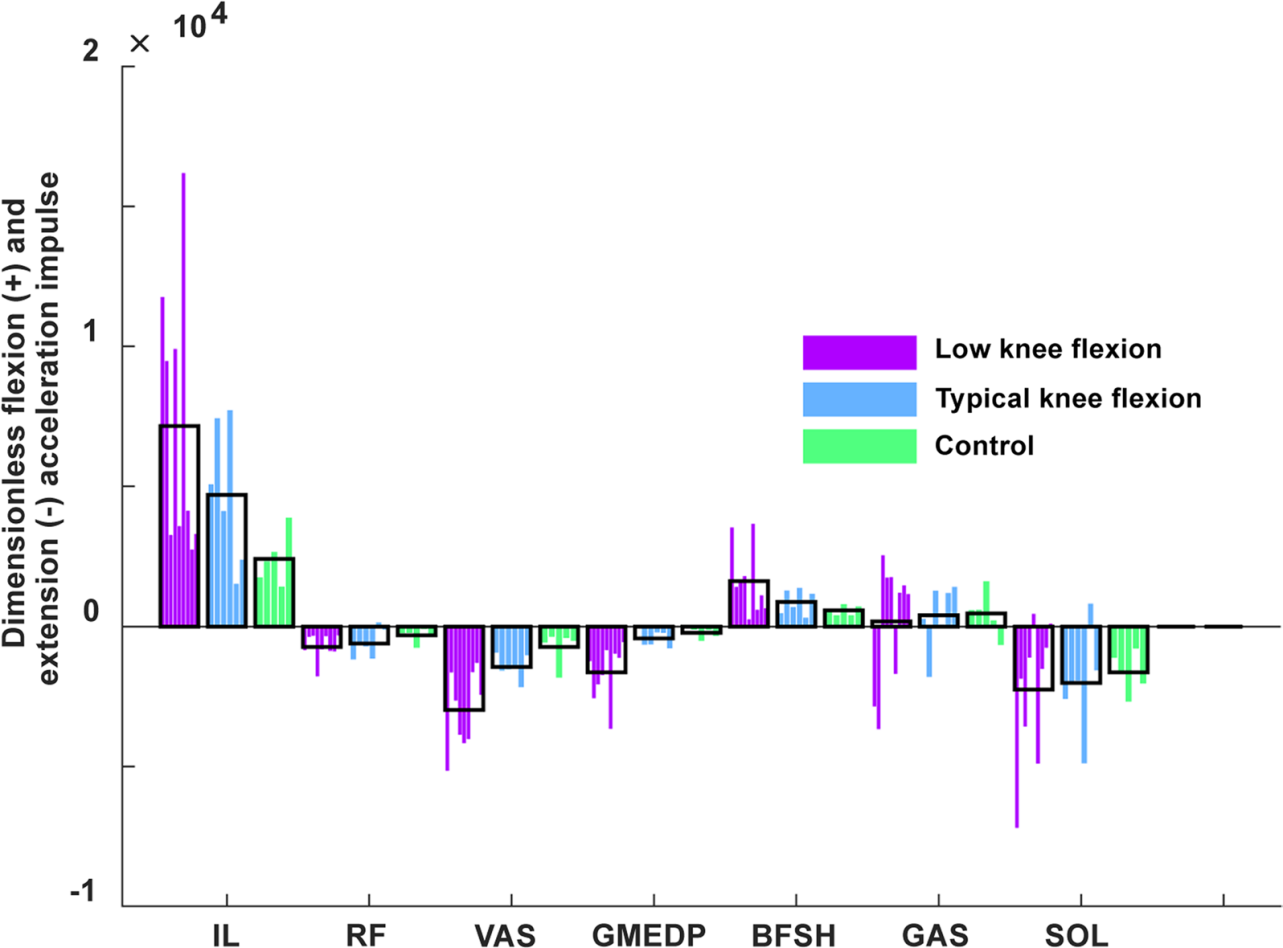
Muscle contributions to knee flexion in pre-swing. Muscle contributions to knee flexion and extension acceleration integrated over pre-swing and normalized by walking speed. Participants are ordered from least to greatest knee flexion during swing

The iliopsoas was the greatest contributor to pre-swing knee flexion acceleration for all groups (Fig. 4). The rectus femoris was not a primary contributor to knee extension acceleration in pre-swing for any group. Rather, the vasti had a greater contribution to knee extension in the low knee flexion group compared to the typical knee flexion and control groups (Fig. 4).

### Leg swing initiation predictors of swing phase knee flexion

**Fig. 5:**
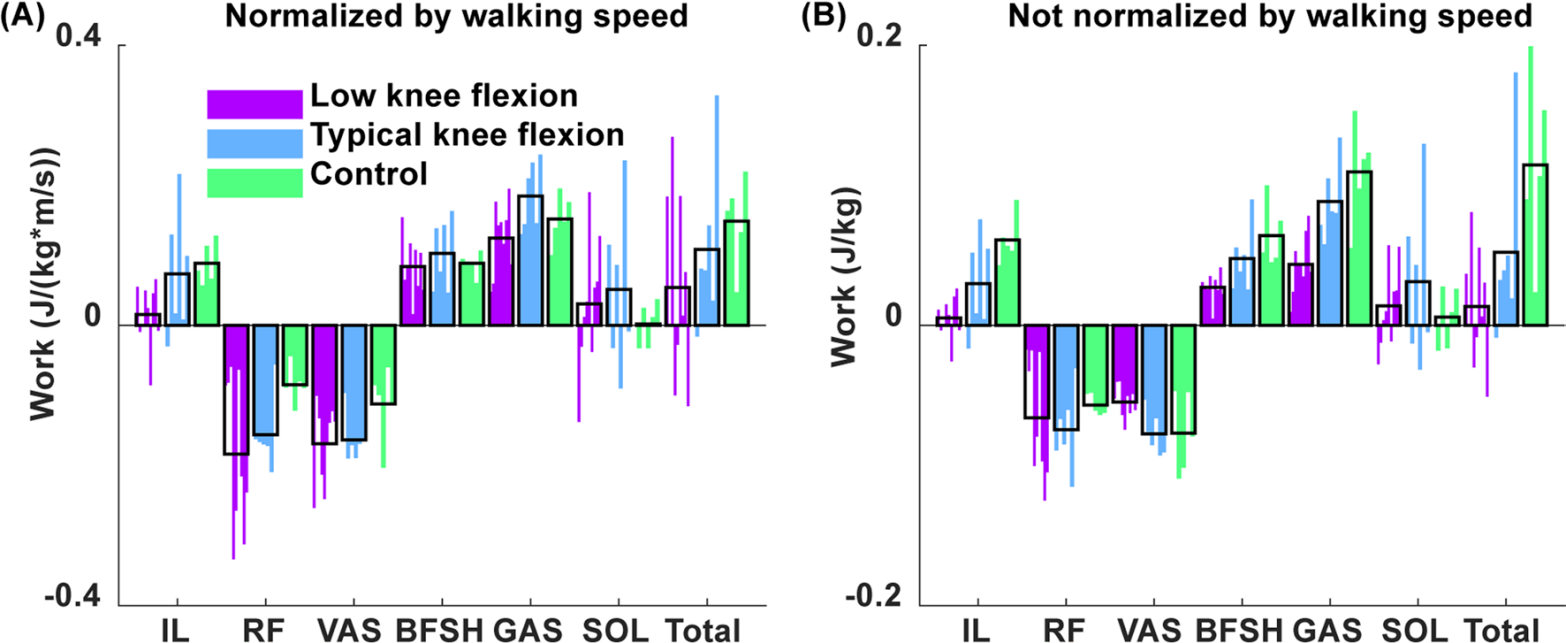
Muscle contributions to leg swing initiation. Musculotendon work performed on the paretic leg in pre-swing **A** normalized by walking speed, and **B** not normalized by walking speed. Participants are ordered from least to most knee flexion during swing

Knee flexion was not predicted by total musculotendon work performed on the paretic leg in pre-swing regardless of whether work was normalized by walking speed (p = 0.18, R^2^ = 0.13, β = 95 and p = 0.58, R^2^ = 0.02, β = 17 for normalized and not normalized, respectively). The low knee flexion group had lower total muscle contributions to paretic leg swing on average, but with high variability between participants (Fig. 5). On average, the low knee flexion group had lower leg swing contributions from the iliopsoas than the typical and control groups regardless of walking speed (Fig. 5).

## Discussion

### Predictors of braking and propulsion asymmetry

The purpose of this study was to investigate impairments in early leg swing that may lead to stiff knee gait in individuals post-stroke. Specifically, we investigated pre-swing muscle contributions to braking, propulsion, knee flexion and leg swing initiation and the underlying relationships between pre-swing muscle function and swing phase knee kinematics in individuals post-stroke. We hypothesized that both (1) plantarflexor contributions to propulsion and (2) vasti contributions to braking would predict propulsion asymmetry. These hypotheses were partially supported. Gastrocnemius contributions to AP GRF predicted propulsion symmetry. However, soleus and vasti contributions to AP GRF impulses did not predict braking or propulsion symmetry (Fig. 1). These results are consistent with previous work showing that in individuals with moderate levels of hemiparetic severity, gastrocnemius and not soleus or vasti EMG activity predicted the AP GRF impulse [20]. In addition, it is well established that the plantarflexor muscles are important rehabilitation targets for improving paretic propulsion and walking speed [15]. For example, Participant 15 produced more paretic propulsion than nonparetic propulsion (PP = 0.71) but had lower net plantarflexor contributions to propulsion on the paretic side throughout stance. This was especially true for the soleus which did not contribute to propulsion, but instead produced braking in early and late stance (Figs. 2C and D), likely due to a lack of leg extension [38]. Participant 15 compensated for these low plantarflexor contributions to propulsion by relying on the hamstrings for propulsion (Fig. 2C and D). However, while paretic propulsion was high relative to the nonparetic leg, Participant 15 had low propulsion overall and thus walked slowly at 0.2 m/s (Table 1), indicating that compensation from the hamstrings is likely not an effective way to produce propulsion. These results can be contrasted with a representative healthy participant (Fig. 2E), who produced braking in early stance with the vasti and propulsion in late stance with the plantarflexors as expected. These results demonstrate that while plantarflexor function is an important predictor of propulsion, paretic propulsion can be attained through compensatory mechanisms, such as from the hamstrings, which cannot be identified through force plate measurements of braking and propulsion.

Although vasti contributions to braking did not predict propulsion asymmetry, it is clear that the vasti can still contribute to propulsion deficits in individuals post-stroke, as extended braking from the vasti was a major contributor to propulsion asymmetry for multiple participants. For example, Participant 2 produced less paretic propulsion than nonparetic propulsion (PP = 0.24) due to prolonged braking from the vasti and rectus femoris despite producing more propulsion with the paretic plantarflexors (Fig. 2A and B). Moreover, we previously showed that individuals with co-activation of the plantarflexors and other muscles such as the vasti had higher paretic braking but not lower propulsion [26], suggesting that propulsion asymmetry occurred not because of low plantarflexor contributions to propulsion but also because of excessive braking from other muscles. Thus, the knee extensor muscles can play a significant role in propulsion asymmetry, as they are primary contributors to braking [9, 39] and can become overactive post-stroke [40]. Vasti that are active in late stance should be an important rehabilitation target, as they can affect both propulsion and knee flexion.

### Braking and propulsion predictors of swing phase knee flexion

We hypothesized that vasti and plantarflexor contributions to propulsion and braking in pre-swing would predict swing phase knee flexion. This hypothesis was partially supported. Lower pre-swing AP GRF impulse and greater vasti contributions to braking predicted less knee flexion in swing, but plantarflexor contributions did not (Fig. 3). Similarly, knee flexion in individuals post-stroke was predicted by late braking forces, but not net propulsive forces [6]. These results can be explained by the fact that the vasti are primary contributors to both braking and knee extension and in individuals post-stroke with stiff knee gait, 83% have been shown to have inappropriate late vasti activity [41]. Other work has suggested that knee flexion deficits may be driven primarily by low ankle push off rather than knee extensors preventing knee flexion [2]. However, those conclusions were developed using a kinematic proxy of push off force (peak vertical acceleration of the malleolus marker), a methodological difference that may explain the differences in our studies. In addition, it was concluded that individuals with lower knee flexion velocity than predicted by the malleolus acceleration model had stiff knee gait due to muscles preventing knee flexion rather than a low push-off force. However, it is likely that excess knee extensor activity could affect vertical malleolus acceleration and therefore these measures are not independent, leading some participants to be classified as having low knee flexion due to low push-off acceleration when knee extensor activity may have also contributed. In summary, our results suggest knee extensor activity in pre-swing predicts reduced knee flexion, while muscle contributions to propulsion in late stance do not.

### Primary contributors to knee flexion and extension in pre-swing

We hypothesized that the rectus femoris would be a major contributor to knee extension in pre-swing in individuals with stiff knee gait. This hypothesis was not supported. Rectus femoris contributions to knee extension were minimal in all groups (Fig. 4), while the stiff knee group had greater knee extension contributions from the vasti. Previous work demonstrated that the while the gluteus maximus, vasti and rectus femoris had the greatest potential to accelerate the knee into extension during pre-swing, the gluteus maximus and rectus femoris produced significantly less force than the vasti in pre-swing, and produced less knee extension than the vasti and soleus, while the iliopsoas produced the most knee flexion [13]. Previous work has also proposed that low knee flexion could be due to weak hip flexor muscles, as hip flexors are key contributors to knee flexion in healthy gait [42, 43]. Modeling studies have identified reduced iliopsoas function during pre-swing in individuals post-stroke with poor walking function [5]. However, we observed that individuals in the low knee flexion group had greater iliopsoas contributions to knee flexion in pre-swing than healthy controls (Fig. 4), although this average was dominated by four individuals, with others having lower than average iliopsoas contributions to knee flexion. This result may have occurred in part because the potential of the iliopsoas to flex the knee increases with reduced knee flexion. Interestingly, while some have found that the gastrocnemius contributed to knee flexion in double support [8, 13, 43], others have found that the gastrocnemius contributed to knee extension in double support [17]. The results of the present study suggest that the gastrocnemius can perform both functions (Fig. 4) depending on the individual’s kinematic state.

There is evidence that the rectus femoris does contribute to stiff knee gait in some but not all cases, as rectus femoris Botox injections for individuals post-stroke improved knee flexion for some individuals but not for those with more severe knee flexion deficits [44]. Thus, there is a need to identify individuals who will benefit from treatments targeting the rectus femoris and others that may experience low knee flexion due to other problems such overactive vasti or weak hip flexors.

### Leg swing initiation predictors of swing phase knee flexion

We hypothesized that greater net musculotendon power delivered to the leg in pre-swing would predict greater knee flexion, but this hypothesis was not supported. While on average the low knee flexion group had less power delivered to the paretic leg in pre-swing compared to the typical knee flexion and control groups, there was significant variability between participants (Fig. 5).

While the rectus femoris was not a primary contributor to knee extension in individuals with low knee flexion, it may still limit leg swing by absorbing power from the leg. Muscles act to either generate, absorb or transfer power between body segments [36, 45]. In healthy individuals, the rectus femoris lengthens in late stance and absorbs power from the leg and redistributes it to the trunk [9]. Compared with healthy controls, a number of participants with low knee flexion had very high power absorption from the rectus femoris in pre-swing (Fig. 5), which would inhibit leg swing initiation and thus limit the trajectory of knee flexion during swing.

The iliopsoas contributed less to leg swing initiation in both stroke groups on average compared to healthy controls, but especially the low knee flexion group (Fig. 5). These results are consistent with previous work showing lower power contributions from the iliopsoas in pre-swing for individuals post-stroke compared to healthy controls [12]. On average the gastrocnemius contributed less to leg swing initiation in the low knee flexion group compared to the typical knee flexion group (Fig. 5), but contributed more to leg swing initiation in the typical knee flexion group when compared to healthy controls. These results of typical knee flexion group contrast previous work that found lower gastrocnemius contributions to leg swing initiation in individuals post-stroke [12]. However, that study only analyzed two representative individuals post-stroke, while ours shows that substantial variability exists between participants. The gastrocnemius may have contributed more to leg swing initiation in the stroke group with typical knee flexion due to the co-contraction seen in some participants (e.g. Participant 2), where greater plantarflexor output was required to overcome excessive braking from the vasti in late stance.

### Limitations

A potential limitation of this study is that individuals post-stroke often have high levels of muscle co-contraction [46, 47], which may be difficult to replicate in simulations using algorithms such as computed muscle control which minimize the sum of muscle activations squared [32]. To address this concern, bilateral EMG was collected and excitations of those muscles were constrained to stay within bounds determined by experimentally collected EMG. Similarly, individuals post-stroke can have altered muscle and tendon properties [22, 23] not taken into account by the model. However, our calculated musculotendon forces and their functional roles are robust to these changes in parameters since our simulation methods identify the necessary net musculotendon forces necessary to reproduce the experimentally measured movement and their function is dictated primarily by the kinematic state of the model which closely tracks with the experimental measurements. Thus, their contributions to biomechanical subtasks should be interpreted as the net effect of both passive and active forces. Other soft tissues such as ligaments can produce passive torques at the joints, but we would not expect these torques contribute significantly to our outcome measures throughout the joint ranges of motion that occur during walking. Soft tissue in human bodies can also oscillate during walking, functioning to store and dissipate energy [48]. However, at these walking speeds we would not expect soft tissue oscillation to be a major contributor to any of our primary outcome measures and thus it would add unnecessary complexity to the model. Another limitation is that this study focused only on muscle contributions to propulsion, while appropriate leg extension angle at push-off also affects propulsion [14, 15, 38]. It is likely that some of these participants experienced propulsion asymmetry in part due to leg positioning and future work should investigate causes of reduced leg extension. A final limitation is the interpretation of results could be affected by some control participants who had below average self-selected walking speeds and asymmetric walking. However, these controls were not used for statistical analyses and served to provide a reference for age-similar individuals without neurological injury rather than perfect walking.

## Conclusions

We observed that some participants had paretic propulsion deficits due to low plantarflexor contributions to propulsion and/or excess vasti contributions to braking. Others appeared to produce sufficient or high paretic propulsion, but accomplished that propulsion via compensatory mechanisms such as a reliance on the hamstrings rather than appropriate plantarflexor activity. Greater vasti contributions to braking in pre-swing predicted lower knee flexion. The rectus femoris and iliopsoas did not directly contribute to lower knee flexion in pre-swing. However, in a number of cases the rectus femoris absorbed more power and the iliopsoas contributed less power from the paretic leg in pre-swing in individuals with low knee flexion. These results highlight the need to identify the underlying causes of propulsion and knee flexion deficits in each individual in order to design more effective rehabilitation strategies.

## Supplementary Information

**Additional file 1: Table S3.** Propulsion and braking symmetry, muscle contributions to propulsion and braking, and knee kinematics. Values for participants in the low knee flexion group are bolded.

## Data Availability

The datasets used in this study are available from the corresponding author on reasonable request.

## Abbreviations

EMG: Electromyography
GRF: Ground reaction force
RRA: Residual reduction algorithm
COM: Center of mass
CMC: Computed muscle control
PP: Percentage paretic propulsion
PB: Percentage paretic braking
AP: Anteroposterior
