# Supplementary material for: Muscle contributions to pre-swing biomechanical tasks influence swing leg mechanics in individuals post-stroke during walking
Source: J Neuroeng Rehabil. 2022 Jun 3;19:55. doi: 10.1186/s12984-022-01029-z (PMC9166530; doi:10.1186/s12984-022-01029-z)
Supplement: Supplementary file 1 — Additional file 1: Table S3. Propulsion and braking symmetry, muscle contributions to propulsion and braking, and knee kinematics. Values for participants in the low knee flexion group are bolded. [file 12984_2022_1029_MOESM1_ESM.pdf]

Table 3: Propulsion and braking symmetry, muscle contributions to propulsion and braking, and knee kinematics. Values for participants in the low knee flexion group are bolded.

Participant	PP	PB	Paretic AP COM acceleration impulse (m/s)			Nonparetic AP COM Acceleration impulse (m/s)			Knee flexion velocity at toe-off (deg/s)	Peak knee flexion (deg)	ROM difference (deg)
			SOL	GAS	VAS	SOL	GAS	VAS			
**1**	**0.24**	**0.51**	**0.64**	**0.205**	**-0.471**	**0.116**	**0.35**	**-0.279**	**122**	**49**	**35**
2	0.31	0.65	0.749	0.328	-0.71	0.225	0.351	-0.238	178	69	12
3	0.42	0.49	0.454	0.348	-0.285	0.142	0.661	-0.196	252	52	12
4	0.52	0.47	-0.176	0.398	-0.156	-0.246	0.457	-0.149	247	71	9
5	0.57	0.49	0.2	0.44	-0.145	0.54	0.235	-0.434	200	61	-9
**6**	**0.24**	**0.72**	**0.259**	**0.308**	**-0.2**	**0.246**	**0.371**	**-0.255**	**93**	**48**	**22**
7	0.5	0.56	0.07	0.401	-0.14	0.252	0.512	-0.38	161	56	1
**8**	**0.55**	**0.64**	**0.648**	**0.232**	**-0.554**	**0.28**	**0.436**	**-0.338**	**-8**	**36**	**42**
**9**	**0.55**	**0.41**	**0.117**	**0.319**	**-0.156**	**0.395**	**0.302**	**-0.274**	**85**	**31**	**31**
**10**	**0.08**	**0.9**	**0.464**	**0.272**	**-0.459**	**0.791**	**0.33**	**-0.573**	**86**	**33**	**21**
**11**	**0.31**	**0.54**	**0.272**	**0.203**	**-0.237**	**0.253**	**0.453**	**-0.241**	**116**	**48**	**20**
12	0.38	0.62	0.288	0.434	-0.222	0.071	0.488	-0.198	185	57	10
**13**	**0.08**	**0.84**	**0.061**	**0.274**	**-0.279**	**0.18**	**0.427**	**-0.357**	**120**	**30**	**24**
**14**	**0.06**	**0.74**	**-0.388**	**-0.266**	**-0.435**	**0.634**	**0.832**	**-0.297**	**158**	**37**	**27**
**15**	**0.71**	**0.41**	**-0.239**	**0.208**	**-0.17**	**0.122**	**0.576**	**-0.509**	**34**	**30**	**21**
**Average**	0.37	0.6	0.228	0.274	-0.308	0.267	0.452	-0.315	135	47	18
**SD**	0.19	0.15	0.322	0.164	0.17	0.241	0.146	0.115	70	13	13
C1	0.55	0.56	-0.077	0.284	-0.065	0.005	0.347	-0.312	253	56	5
C2	0.53	0.52	0.079	0.3	-0.26	0.262	0.376	-0.293	338	68	3
C3	0.43	0.55	-0.067	0.251	-0.22	0.417	0.208	-0.462	363	77	-1
C4	0.72	0.33	0.118	0.322	-0.225	0.158	0.483	-0.337	259	72	-9
C5	0.56	0.47	0.221	0.381	-0.295	0.279	0.384	-0.379	335	72	-7
**Average**	0.56	0.48	0.055	0.307	-0.213	0.224	0.36	-0.357	310	69	-2
**SD**	0.09	0.08	0.114	0.043	0.079	0.137	0.088	0.06	45	7	6
